# Using Bayesian-PBPK modeling for assessment of inter-individual variability and subgroup stratification

**DOI:** 10.1186/2193-9616-1-6

**Published:** 2013-04-11

**Authors:** Markus Krauss, Rolf Burghaus, Jörg Lippert, Mikko Niemi, Pertti Neuvonen, Andreas Schuppert, Stefan Willmann, Lars Kuepfer, Linus Görlitz

**Affiliations:** 1 Bayer Technology Services GmbH, Computational Systems Biology, Leverkusen, 51368 Germany; 2 RWTH Aachen, Schinkelstr, Aachen Institute for Advanced Study in Computational Engineering Sciences, Aachen, 2, 52062 Germany; 3 Clinical Pharmacometrics, Bayer Pharma AG, Wuppertal, 42117 Germany; 4 Department of Clinical Pharmacology, University of Helsinki, Helsinki, Finland; 5 HUSLAB, Helsinki University Central Hospital, Helsinki, Finland

**Keywords:** Physiologically-based pharmacokinetic modeling, Bayesian approaches, Markov chain Monte Carlo, Inter-individual variability, OATP1B1, Drug development, Pravastatin

## Abstract

**Purpose:**

Inter-individual variability in clinical endpoints and occurrence of potentially severe adverse effects represent an enormous challenge in drug development at all phases of (pre-)clinical research. To ensure patient safety it is important to identify adverse events or critical subgroups within the population as early as possible. Hence, a comprehensive understanding of the processes governing pharmacokinetics and pharmacodynamics is of utmost importance. In this paper we combine Bayesian statistics with detailed mechanistic physiologically-based pharmacokinetic (PBPK) models. On the example of pravastatin we demonstrate that this combination provides a powerful tool to investigate inter-individual variability in groups of patients and to identify clinically relevant homogenous subgroups in an unsupervised approach. Since PBPK models allow the identification of physiological, drug-specific and genotype-specific knowledge separately, our approach supports knowledge-based extrapolation to other drugs or populations.

**Methods:**

PBPK models are based on generic distribution models and extensive collections of physiological parameters and allow a mechanistic investigation of drug distribution and drug action. To systematically account for parameter variability within patient populations, a Bayesian-PBPK approach is developed rigorously quantifying the probability of a parameter given the amount of information contained in the measured data. Since these parameter distributions are high-dimensional, a Markov chain Monte Carlo algorithm is used, where the physiological and drug-specific parameters are considered in separate blocks.

**Results:**

Considering pravastatin pharmacokinetics as an application example, Bayesian-PBPK is used to investigate inter-individual variability in a cohort of 10 patients. Correlation analyses infer structural information about the PBPK model. Moreover, homogeneous subpopulations are identified *a posteriori* by examining the parameter distributions, which can even be assigned to a polymorphism in the hepatic organ anion transporter OATP1B1.

**Conclusions:**

The presented Bayesian-PBPK approach systematically characterizes inter-individual variability within a population by updating prior knowledge about physiological parameters with new experimental data. Moreover, clinically relevant homogeneous subpopulations can be mechanistically identified. The large scale PBPK model separates physiological and drug-specific knowledge which allows, in combination with Bayesian approaches, the iterative assessment of specific populations by integrating information from several drugs.

**Electronic supplementary material:**

The online version of this article (doi:10.1186/2193-9616-1-6) contains supplementary material, which is available to authorized users.

## Background

Tailor-made therapeutic designs require a functional understanding of the processes governing the distribution of substances within an organism. Anthropometric parameters like age or weight have great influence on the level of drug exposure in the human body (Willmann et al., 2007). Furthermore, the genetic predisposition of a patient is very important, since different genotypes can have significant effects on drug metabolization processes (Eissing et al., 2012; Lippert et al., 2012). In the worst case, side effects due to increased (off-)target tissue drug concentrations become critical for patient safety (Lippert at al., 2012). The early identification of subgroups showing significantly increased adverse event rates is a difficult task since only limited information about a new drug is available but is of utmost importance to prevent costly drug withdrawals in later phases of the drug development process (Kuepfer et al., 2012). Therefore, a mechanistic understanding of pharmacokinetics (PK) is essential in drug development to optimize the risk-benefit profile of a drug. This involves in particular the identification of high-risk subgroups in which an unfortunate combination of predisposition and non-optimal dosing schemes lead to potentially life-threatening side effects. In clinical practice, such subgroups have to be treated with individualized dosing schemes, which need to be designed and surveyed with adequate diagnostics.

The amount and complexity of preclinical and clinical data generated along the drug development process usually represents an immense challenge for the generation of an in-depth mechanistic understanding. Here, *in silico* approaches provide a rational and efficient way to aggregate all data for the determination of drug PK and pharmacodynamics (PD) in support of the drug development process. Once established and validated, computational models allow a detailed analysis of the effect of different dosing schemes or varying anthropometry or physiology by simulating the behavior of a drug in the body. In contrast to the rather descriptive consideration of PK and PD in classical compartmental approaches (Meibohm & Derendorf, 1997), physiologically-based pharmacokinetic (PBPK) models are based on a large amount of prior physiological and anthropometric information which is integrated in the model structure (Nestorov, 2007; Rowland et al., 2011; Schmitt & Willmann, 2004), Since PBPK models explicitly distinguish between properties of the compound and properties of the patients, respectively, they allow separation of physiological and drug-induced effects. Generally, such models consist of several compartments, describing the organs, which are further on subdivided in more detailed submodules such as interstitial, intracellular or vascular space. Starting from models with only few equations (Pang & Durk, 2010), they exist on all levels of complexity, up to more than one hundred ordinary differential equations (ODEs) and hundreds of parameters (Eissing et al., 2011; Willmann et al., 2003a). PBPK models have previously been used for mechanistic analyses of drug PK (Meyer et al., 2012), pharmacogenomics (Eissing et al., 2012), multiscale modeling (Krauss et al., 2012) or analysis of rare adverse events (Lippert et al., 2012; Willmann et al., 2009). However, current use of such models often provides only a single value time-concentration curve, describing the behavior of a mean patient, neglecting potentially relevant individual properties. This is even more severe as PBPK models allow the creation of personalized models for individual patients by explicitly representing the individual physiological parameters. Thereby it is possible to mechanistically describe special populations (Edginton & Willmann, 2008) or genetic predisposition of patients in pharmacogenomics applications (Eissing et al., 2012; Swen et al., 2007). Nevertheless, PBPK models frequently lack the rigorous quantification of inter-individual variability in parameters which cannot be derived from the patients’ anthropometry.

Up to now, population simulations try to assess inter-individual variability in PK in groups of patients (Schüttler & Ihmsen, 2000; Willmann et al., 2007). This is examined by *a priori* variation of physiological parameters and cross correlations to other model parameters. Such correlations are estimated by means of scaling laws depending on the anthropometry, since no literature information on inter-individual variability of organ weights or blood flows is available (Willmann et al., 2007). Therefore, since the physiology of every individual is calculated *before* the simulation, such population simulations cannot be processed if for example special groups of patients are investigated where little prior information about their anthropometry, (patho-)physiology or genotype-phenotype correlation is available.

An alternative approach to analyze the inter-individual variability and to perform population simulations is Bayesian modeling (Bolstadt, 2010). Bayesian statistics is based on Bayes’ theorem, which provides a rational way to combine prior information on parameters with the information contained in data to infer the variability of parameters and therefore also the PK variability *a posteriori*, even with little prior information. The key idea of Bayesian statistics is to define unknown parameters as random variables, which is in contrast to the general approach in statistics, where parameters are defined as fixed, but unknown constants. In Bayes’ theorem, prior knowledge about the parameters is updated with new experimental data in the so-called posterior distribution (Bolstadt, 2010). Determining the posterior distribution explicitly is very difficult or even impossible with nonlinear model kernels or when many parameters are considered simultaneously. In such cases, Markov-chain Monte-Carlo (MCMC) methods can be used to estimate the posterior distribution.

MCMC covers a large group of algorithms containing for example usual (Geman & Geman, 1984; Hastings, 1970; Metropolis, 1953), adaptive (Atchadé & Rosenthal, 2005; Gilks et al., 1998; Haario et al., 2001; Roberts & Rosenthal, 2009) and particle (Andrieu et al.,^2010^) MCMC approaches to take samples from the posterior distribution of a parameter vector. The core idea of MCMC is to sample the unknown variables along a Markov chain, which has the posterior distribution as its stationary distribution. If several parameters are considered, such probability distributions are high dimensional. Thorough analysis of this posterior distribution quantifies inter-individual variability of a group of patients as well as the co-variability of the parameters, allowing the identification of homogenous subgroups. Bayesian approaches have already been used in conjunction with PBPK modeling, especially in toxicological questions (Bernillon & Bois, 2000; Bois et al., 2010), but also for population PK (Gelman et al., 1996; Gueorguieva et al., 2006; Yang et al., 2009). However, often the PBPK models used have been comparatively small and have contained lumped parameters carrying mixed information of different physiological or drug specific parameters. By using a large scale PBPK model, which separates drug specific from population specific information, in combination with Bayesian approaches, an iterative characterization of special populations by optimally leveraging information from different drugs can be achieved.

**Figure 1:**
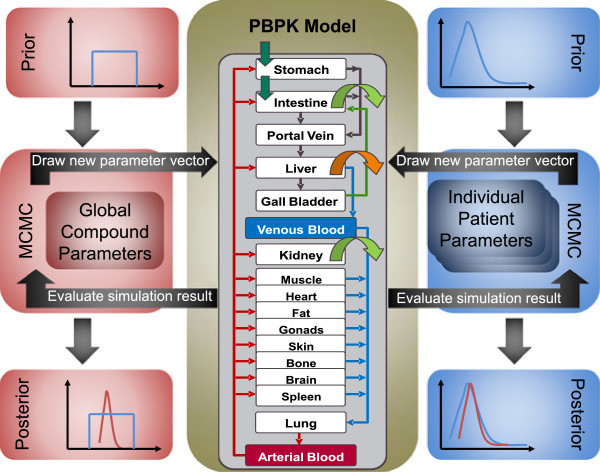
**Schematic representation of the combined Bayesian-PBPK approach.** A block-wise Metropolis-Hastings Markov chain Monte Carlo algorithm was used to sample the posterior distribution of individual patients’ physiology on the one hand and global compound parameters on the other hand. The underlying model kernel was provided by detailed mechanistic physiologically-based pharmacokinetic models.

In this work, we present a new approach applying MCMC to Bayesian-PBPK modeling for the assessment of inter-individual variability in groups of patients (Figure 1). Notably, we use a highly detailed and mechanistic PBPK model, where every organ is divided into four sub-compartments describing the intracellular space, the interstitial space, the blood plasma and the blood cells (Willmann et al., 2003a). Due to a segregated representation of the physiology of the patient and the underlying distribution model which is related to the physicochemistry of the drug, physiological parameters, genotype-specific (in the following also referred to as physiological parameters) and drug-specific parameters, respectively, can be considered separately. This model representation together with Bayesian approaches allows direct inference of physiological and drug-specific information, such as the variability in organ volumes or the uncertainty in the lipophilicity of a drug. Therefore, the main sources of variability within the PK of a drug may be quantified by analyzing the posterior distribution. Moreover, we present a way to analyze the posterior to identify clinically relevant homogenous subgroups, such as patients with a specific genotype linking to a PK (or PD) phenotype. Additionally, the use of such a mechanistic model bears great extrapolation capacity, by enabling an iterative use of the posterior as the prior distribution of a new run. Since physiology and drug are treated independently, posterior physiological information of a run with a known drug can be used for the investigation of a new drug candidate in the same group of patients such that physiological knowledge is conserved. The same applies for using the same drug in different populations. This allows for example the construction of a large database, wherein prior information about physiological as well as drug-specific parameters may be updated with experimental data of lots of experiments. Since only little literature information about specific parameters is available, informative prior distributions can be ‘learned’ after several MCMC runs with different experimental data.

Taken together, besides the assessment of inter-individual variability and co-variability of physiological parameters, our presented approach additionally provides a very valuable tool for long-term characterization of special populations as well as drug physicochemistry.

## Methods

### Physiologically-based pharmacokinetic modeling

PBPK models quantitatively consider the absorption, distribution, metabolization and excretion (ADME) of exogenous and endogenous substances at a very high level of detail (Nestorov, 2007; Rowland et al., 2011; Schmitt & Willmann, 2004; Willmann et al., 2003a). They mechanistically describe all relevant processes based on a large amount of prior physiological information. The models consist of compartmental representations of all relevant organs, tissues and the vascular system. The underlying model structure which is based on generic distribution models quantifies the mass transfer between the vascular system and the organs (Poulin et al., 2001; Rodgers et al., 2005; Rodgers & Rowland, 2006; Willmann et al., 2003b; Willmann et al., 2004). Parameters in the PBPK model can be divided into two types of parameters: (1) physiological parameters such as organ volumes or blood flow rates which are obtained from large collections of physiological data integrated into the PBPK software database and (2) substance-specific parameters describing the physicochemistry of a compound such as the molecular weight or the lipophilicity. Moreover, the large amount of prior physiological information constraints the number of independent parameters in the PBPK model which need to be identified (usually less than ten). The PBPK model of the present work consists of more than one hundred ordinary differential equations containing hundreds of parameters. The clear separation of physiology and drug-specific parameters due to the mechanism-based approach and the size of the model also allow the separated inference in parameter-identification processes.

The pravastatin model considered in this work was built with the software tools PK-Sim and MoBi. Academic licenses for both tools are available free of charge and both PK-Sim and MoBi have been explained in detail before (Eissing et al., 2011; Willmann et al., 2003a). The anthropometric information of the patients regarding age, weight and height further specifies the selection of physiological parameters as provided in the software, which allows a specific parameterization of the PBPK model.

### Bayesian approach in combination with PBPK modeling

PBPK models can be parameterized for individuals with defined anthropometries such as age, sex, weight or height. Nevertheless, every model represents a mean value model, assuming that a group of individuals with the same anthropometry also has the same parameterization. However, even in-between defined groups of patients, parameters such as organ volumes or blood flow rates, can show substantial variation from individual to individual. Additionally, the determination of substance-specific parameters often contains uncertainties since such parameters are determined *ex vivo*. Thus, substance-specific parameters also vary, but in contrast to the individual parameters, their value is the same for all patients. We call this type of parameter global parameters, in contrast to the parameters we call individual parameters, which need to be randomized separately in every patient.

Both, variability and uncertainty in individual parameters *θ*^*I*^ and uncertainty in global parameters *θ*^*G*^ influence the PK of endogenous and exogenous compounds in an organism. Uncertainty can be reduced for example by increasing the number of experiments or by an optimized experimental design. In contrast, inter-individual variability is a characteristic property and cannot be reduced (Bernillon & Bois, 2000).

But how to determine such uncertainty and variability in a group of patients? Classical parameter identification relies on optimization-based approaches. They determine only *the* parameter vector with the highest probability to fit to the data, called maximum likelihood estimator. In contrast, Bayesian approaches aim for the identification of a probability distribution of the parameter vector. Furthermore, they also consider prior knowledge about the parameters, and ‘update’ this prior knowledge by integration of new experimental data based on Bayes’ theorem given by1

The posterior distribution *p(θ|D)* combines prior knowledge *p(θ)* about the parameter vector *θ* (*θ∈ℝ*^*P×1*^) with the likelihood function *p(D|θ).* The likelihood function represents the closeness to the data *D*, where *D={x*_*i,k*_*,t*_*i,k*_*} with i=1,…,N*_*k*_*. x*_*i,k*_ represents the measurement of individual *k (k=1,…,K)* at time point *t*_*i,k*_. The idea of Bayesian approaches is to model any unknown parameter as random variable, since the true value of the parameter is unknown. However, especially in high dimensional problems, the numerical determination of the posterior is almost impossible. Therefore, several methods have been developed which draw a sample from the posterior distribution to estimate its probability density. Markov chain Monte Carlo (MCMC) approaches are a large group of sampling algorithms which can be divided to a great extent into two groups: Metropolis-Hastings (MH) algorithms (Hastings, 1970; Metropolis, 1953) and the Gibbs sampler (Gelfand & Smith, 1990; Geman & Geman, 1984). In contrast to classical Monte Carlo sampling, MCMC samples from a special Markov chain which, in our case, is constructed to have the posterior distribution as its long-run stationary distribution (Andrieu et al., 2003).

For our combined Bayesian-PBPK approach, we considered a block-wise MH algorithm to sample from the posterior distribution. In contrast to a single MH block containing all parameters, dividing the parameter space in blocks improves the convergence speed of the Markov chain. Thus, one MH step was applied to every block of parameters, conditional on knowing the other parameter values which were not in this block. We considered K+1 main blocks, one for every individual and one containing the global parameters. One MH step was performed as follows:

i. Let *θ*^*I*^_*k*_*(n)* be the parameter vector of the individual parameters of individual k after n steps. Propose a new parameter vector *θ*^*I*^_*k*_*’* by random sampling from proposal density *Q(θ*^*I*^_*k*_*(n),∙ )*.
ii. Generate *u∈[0,1]* uniformly distributed. Examine if2
iii. If true: *θ*^*I*^_*k*_*(n+1) = θ*^*I*^_*k*_*’*, else: *θ*^*I*^_*k*_*(n+1)= θ*^*I*^_*k*_*(n)*; => *θ*_*k*_*(n+1)=[θ*^*I*^_*k*_*(n+1), θ*^*G*^*(n)]*.

In another MH block, only the global parameters *θ*^*G*^ were sampled and the individual parameters *θ*^*I*^_*k*_*(n)* were fixed to the value of the last individual MH step:

i. Let *θ*^*G*^*(n)* be the parameter vector of the global parameters after n steps. Propose a new parameter vector *θ*^*G*^*’* by random sampling from proposal density *Q(θ*^*G*^*,∙ )*.
ii. Generate *u∈[0,1]* uniformly distributed. Examine if3
iii. If true: *θ*^*G*^*(n+1)= θ*^*G*^*’*, else: *θ*^*G*^*(n+1)= θ*^*G*^*(n)*; => *θ*_*k*_*(n+1)=[θ*^*I*^_*k*_*(n), θ*^*G*^*(n+1)]*.

Notably, a truncated normal distribution centered around the current value *θ* was chosen for the proposal density *Q(θ,∙ )*, since sampling was constrained by physiological constraints (*θ*_*min*_*, θ*_*max*_) of each parameter.

For every individual *k*, informative prior distributions were defined as lognormal distributions for every of *M* parameters for which enough literature information about the population wide distribution was available (Gelman et al. 1996). Otherwise, flat non-informative prior distributions (for parameters *P-M*, remember that *θ∈ℝ*^*P×1*^) were chosen:4567

The uncertainties *σ*_*k*_ represented the measurement error of the experimental data *D*. Since their original values were unknown they were also considered as individual-specific random variables and have been assigned a prior distribution. Notably, the prior distributions were assumed to be independent from each other, since no information about co-variances was available at the beginning. However, after several runs with the same population as described in the introduction, prior distributions may be updated with the information about the co-variances between the parameters.

The likelihood function was defined with the help of a least squares error model. Additionally it was assumed that errors were distributed normal on a log-scale and were independent.8

*f*^*k*^*(θ*_*k*_*,t*_*i,k*_*)* represents the time-resolved evaluation of the PBPK model with the respective parameter vector *θ*_*k*_.

### Results: Inter-individual variability in pravastatin pharmacokinetics

As an application example for the presented Bayesian-PBPK approach we here considered the PK of the 3-hydroxy-3-methyl-glutaryl-CoA (HMG-CoA) reductase inhibitor pravastatin. This drug has been known for long and its genotype mediated inter-individual variability is well-characterized (Everett et al., 1991; Kivisto & Niemi, 2007; Serajuddin et al., 1991; Singhvi et al., 1990). Therefore, this case study is well suited to demonstrate the advantages of our approach, in particular the assessment of inter-individual variability as well as the identification of homogenous subgroups even with small amounts of experimental data.

Pravastatin is a HMG-CoA reductase inhibitor which lowers the cholesterol level within the body and thereby prevents cardiovascular diseases. Compared to other statins, it has a low lipophilicity (Serajuddin et al., 1991) such that pravastatin uptake is mainly distributed by active transporters (Kivisto & Niemi, 2007): On the one hand, the organic anion transporting polypeptide (OATP1B1) transports pravastatin into the intracellular space of the liver and on the other hand the organic anion transporter 3 (OAT3) inserts pravastatin in the intracellular space of the kidneys (Kivisto & Niemi, 2007). In the liver, pravastatin is excreted by biliary excretion, leading to enterohepatic circulation, while tubular secretion is the main pathway to excrete pravastatin from the kidneys (Hatanaka, 2000). Thereby, both routes of excretion are also performed by an active transporter, the multidrug resistance-associated protein 2 (MRP2) (Additional file 1: Figure S1). MRP2 is also significantly expressed in the apical membrane of enterocytes in the duodenum and jejunum. The bioavailability of pravastatin is low due to an incomplete absorption in the small-intestine (Kivisto & Niemi, 2007).

Notably, significant alterations in pravastatin PK are associated to three different genotypes (SNP; c.521T→C, p.Val174Ala) of SLCO1B1 encoding for OATP1B1 (Kivisto & Niemi, 2007; Niemi et al., 2006). This genotype determines the transporter activity (Lippert et al., 2012); the CC genotype has decreased activity compared to the normal TT genotype, which leads to higher pravastatin concentrations in the body. In contrast, no such effect is known for MRP2.

For our analyses we considered a previously established and validated PBPK model of pravastatin (Lippert et al., 2012) for an oral dose of 40 mg. In this model, active transport processes have been established in the interstitial (OATP1B1) and the intracellular space (MRP2) of the liver as well as in the interstitial space of the kidneys (OAT3). Additionally, MRP2 mediated transport was considered in the gastrointestinal compartment of our model as well as the intracellular space of the kidneys. Tissue specific enzyme activity was estimated by using gene expression data as a proxy for protein abundance. Notably, this allows the discrimination between organ-specific protein levels and the global catalytic rate constant *k*_*cat*_ (Meyer et al., 2012). A luminal clearance reaction in the small intestine accounted for the low bioavailability of pravastatin.

**Figure 2:**
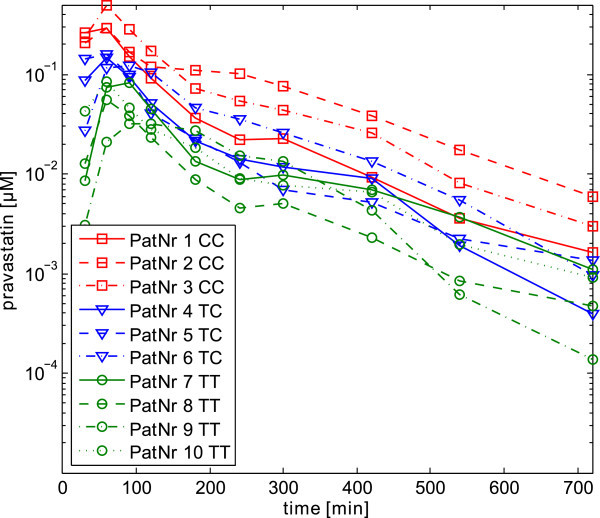
**Experimental data of ten patients which were considered for the assessment of their inter-individual variability.** The patients have been chosen out of a dataset of 32 patients provided by Niemi et al. (Niemi et al., 2006) such that all three possible genotypes of the hepatic uptake transporter OATP1B1 occurred equally.

The experimental data was provided from previously published studies (Niemi et al., 2006). Out of the dataset of 32 patients, 10 patients have been chosen randomly to lower computational costs (Figure 2). Nevertheless, the three genotypes of OATP1B1 are distributed equally in the chosen population. To describe the variability in all relevant ADME processes, 8 individual parameters together with 4 global parameters were chosen for the Bayesian analysis (Table 1), which means the variation of 84 parameters in total. During the separation of the parameters into different blocks, it is very important to know if parameters are correlated, since correlated parameters have to be sampled in one block (Smith et al., 1992). Our block structure is driven by the clear separation between substance and individual physiology in the PBPK model, therefore, we can assume that all parameters of different blocks are independent and uncorrelated (see also the discussion)and we can assure that no lumped parameters exist which depend on physiological and substance-specific information.

**Table 1:** **Parameters to be varied in the coupled Bayesian-PBPK approach**

Parameter	Unit	Abbreviation	Type
Intestinal permeability	cm/min	P_int_	Individual
Intestinal transit time	min	ITT	Individual
Gastric emptying time	min	GET	Individual
Luminal clearance factor	μM/min	CL_lum_	Individual
k_cat_ OATP1B1 (factor)	-	k_cat,O_	Individual
k_cat_ MRP2 (factor)	-	k_cat,M_	Individual
Lag Time of enterohepatic circulation	min	EHClagtime	Individual
Measurement error	-	sd	Individual
Lipophilicity (logP)	-	lip	Global
Unbound protein fraction	%	fu	Global
Km OATP1B1	μM	Km_O_	Global
Km MRP2	μM	Km_M_	Global

With the established PBPK model, the combined Bayesian-PBPK approach was processed and 300000 iteration steps were calculated. The computation time was 3.6 s/iteration and was performed on a quad core i5 processor running under Windows 7. Although the process is independent from the initial guesses, parameter start values have been estimated with the help of an identification process with a single patient to reduce convergence time (Additional file 2: Table S1).

**Figure 3:**
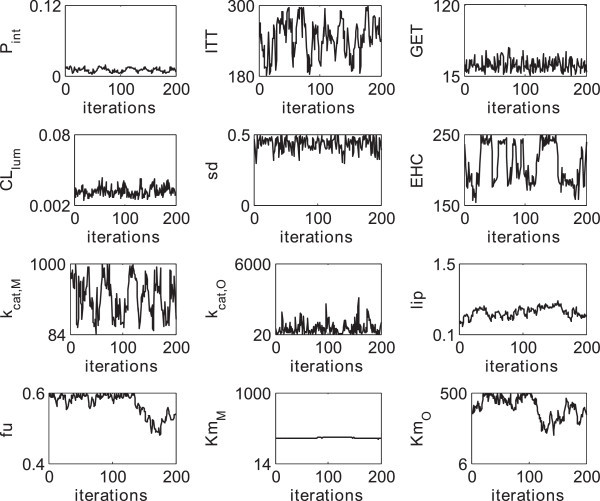
**Exemplary representation of a subsample of the posterior distribution.** After a burn-in period of 150000 steps, a subsample of 200 parameter vectors was drawn for each patient. The figure shows the traces for all eight individual parameters exemplarily for one patient as well as the four global parameters which were the same for all patients. The limits on the y-axis represent the physiological constraints (*θ*_*min*_ *, θ*_*max*_).

During the first 150000 steps the parameter vectors have not been sampled from the correct distribution. For this so-called burn-in period the samples were discarded. By subsampling 200 parameter vectors of each patient from the remaining 150000 steps, an independent sample of the posterior distribution was drawn. The resulting traces are exemplarily shown for one patient (Figure 3). Notably, the four global parameters remain the same for every patient as described above, since they only depend on the physicochemistry of the drug.

**Figure 4:**
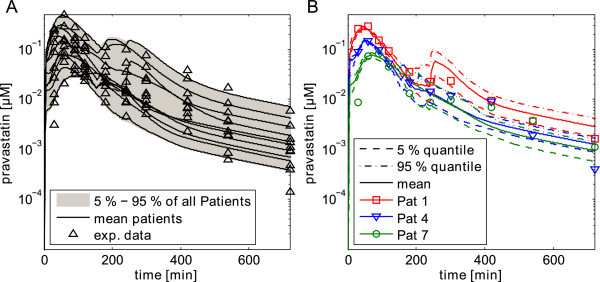
**Inter-individual variability of pravastatin pharmacokinetics.** (**A**) Simulations were performed for each patient, simulating the pravastatin PBPK model with each of the 200 parameter vectors which were subsampled out of the posterior distribution. Next, the 5–95% quantile was calculated over all patients (with all 2000 samples) and plotted. Additionally, the mean value PK curve was monitored for every patient together with the experimental data. (**B**) Simulations were performed for three exemplary patients by simulating the pravastatin model with each of the 200 parameter vectors which were subsampled out of the posterior distribution. The 5% and 95% quantiles were calculated and plotted out of the respective subsample for each patient, together with the mean value curve and the experimental data.

**Figure 5:**
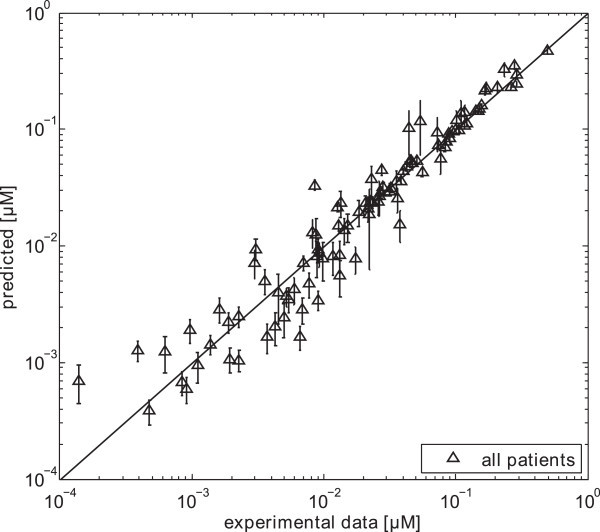
**Correlation between predicted mean values and experimental data.** Mean concentration values at the same time points as the experimental data were monitored for all patients.

**Figure 6:**
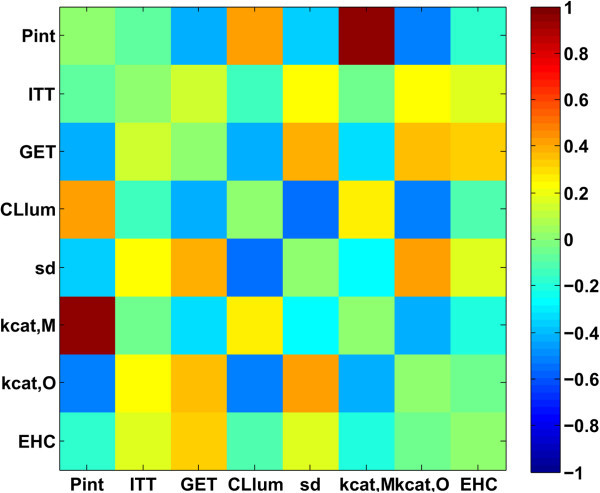
**Correlation matrix of all individual parameters.** Spearman correlation coefficients were calculated from the overall subsample of 2000 parameter vectors for all parameter combinations to identify structural connections. To improve the visualization of the correlations the main diagonal was set to zero.

Next, the PK which described the inter-individual variability of the whole population and the mean PK of the corresponding patients were simulated (Figure 4A). The inter-individual variability was estimated by calculating the 5–95% range of all patients. To demonstrate that the depicted inter-individual variability did not already result from large variability and uncertainty of the single patients, the 5% and 95% quantiles and mean values for three exemplary patients were illustrated (Figure 4B). Additionally, the patient-specific mean value curves show good agreement to the experimental data (Figure 5). Notably, beside the PK range which is kind of a ‘macroscopic’ result of the posterior parameter distribution a lot of other information can be obtained by directly analyzing the posterior. The calculation of correlations between the 8 individual parameters provided information about dependencies between the various parameters in the model. For example, a strong correlation between *Pint* and *k*_*cat,M*_ was observed (Figure 6).

We next asked whether our approach can also be used for the identification of specific subgroups within a population. This is a challenging task in particular in early phases of drug development, since only little prior knowledge may be available. Therefore, we asked if our Bayesian-PBPK approach enabled the identification of such homogenous groups of patients even if no additional information was taken into account and considered the transporter activities of MRP2 and OATP1B1 as a putative source for subgroup stratification.

**Figure 7:**
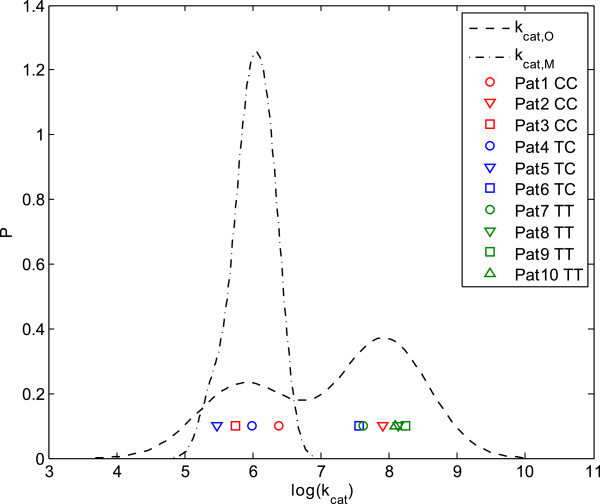
**Identification and assignment of patient subgroups by monitoring the logarithmic mean for each patient.** A density estimation of the logarithmic mean values supported the identification of specific patient subgroups. The logarithmic mean values of the transporter activities for MRP2 and OATP1B1 were calculated from the subsample of the posterior and the kernel densities were quantified. Since the density for OATP1B1 provided the separation of the patient logarithmic mean values into two groups, single values were also plotted with symbols. Additionally, they were colored related to their specific genotype.

First, we performed a Shapiro-Wilk test for normal distribution (Shapiro & Wilk, 1965) of the logarithmic mean values of the 200 samples of every patient, since protein expression has to be log-normally distributed in homogenous groups of patients (Sigal et al., 2006; Spencer et al., 2009). The results supported the hypothesis of lognormal distribution for MRP2 (p>0.75) and gave a strong indication of rejection of the hypothesis for OATP1B1 (p<0.1). Visual inspection of the estimated kernel densities (Bowman & Azzalini, 1997) of the logarithmic mean values (Figure 7) supported this, since two groups of patients were monitored for OATP1B1 but the density of MRP2 is clearly normally distributed. Thus, with regard to OATP1B1 the patient mean values were analyzed individually to examine which patient can be assessed to which group (Figure 7). A clear separation into two groups of four and six patients, respectively, was found. It should be noted that this separation of the OATP1B1 transporter activity was not an implicit property of the model structure but emerged as a result during the Bayesian-PBPK approach.

This grouping of patients was compared to the different genotypes in OATP1B1, which is known to significantly influence PK. This consideration led to a clear separation of the two homozygous genotypes, which demonstrated the capability of the approach to give strong hints about the reasons for subgroup stratification, even when only little experimental data of a small population was available.

### Discussion

In the present work, we introduced a combined Bayesian-PBPK approach to quantify inter-individual variability in groups of patients. In former work such approaches have been mainly used in the context of toxicokinetics (Bois et al., 2010; Jonsson & Johanson, 2002). For PK simulations in virtual groups of patients, usually PBPK population models have been considered (Willmann et al., 2007). One drawback of combined Bayesian approaches so far has been that many PBPK models were relatively small and not fully physiology-based (Willmann et al., 2007). Due to model reduction processes, parameters contain mixed information about the patients’ physiology and the substance. This ambiguity in parameter information prevents extrapolation to other drugs or groups of individuals. In contrast, the here used detailed mechanistic PBPK model is fully physiology-based and substance-specific parameters, physiological parameters and genotype-specific parameters are considered separately. The consideration of a Bayesian approach in combination with such models enables the inference of both physiological variations in a population and intra-individual parameter uncertainty of single patients and in contrast to the PBPK population approaches even when little experimental data is available.

Generally, one challenge in the performance of the approach is the identification of the convergence of the Markov chain. To prove that a finite sample of the posterior is representative to the posterior distribution, several tools have been developed. Most of them, however, are very difficult to use and have a relatively high probability to fail (Cowles & Carlin, 1996). Moreover, they were developed for less complex models and of lower dimensionality. To decide after how many steps the burn-in period ended, we visually inspected the traces of the parameters in all patients. Nevertheless, since this is a crucial point in MCMC, high quality convergence analyses should be considered in future work.

The gold standard for the assessment of inter-individual variability would be the identification of whole patients’ physiology and the integration of as much experimental data as possible. In our model this would lead to the identification of hundreds of parameters per patient and thousands of parameters for a large population. Due to computational restrictions we here chose only a population of 10 patients and varied only several parameters per patient, however, the parameters have been chosen in a way such that all the important ADME processes were represented. A possible concept for computational reduction would be the parallelization of the individual Metropolis-Hastings blocks, which would reduce the computation time by the number of patients if enough computational power is available. Notably, the presented concept is not constrained in its dimensionality, therefore also the investigation of large populations and hundreds of parameter is possible, which provides great opportunities for the assessment of inter-individual variability in clinical trials.

By using a block-wise MH algorithm, a standard MCMC algorithm was chosen for this combined Bayesian-PBPK approach. This enabled first analyses of the behavior of the results as well as the simulation process itself under consideration of large mechanistic PBPK models. The several MH blocks allowed the separation between individual parameters and global parameters and reduced the convergence time of the run since every block could converge faster as if all parameters would have been varied in one large block. In following investigations, different algorithms such as adaptive approaches (Gilks et al., 1998; Haario et al., 2005; Roberts & Rosenthal, 2009) could be tested to identify the ones which for example further reduce convergence time or improve the mixing of the Markov chains. Furthermore, the use of Bayesian population approaches could be an option to make better inferences about the whole population, especially when only few patients are considered (Bernillon & Bois, 2000; Bois et al., 2010).

Concerning the global parameters it has to be noted that such parameters have to be chosen very carefully, since they have by definition a large effect on all obtained individuals. In our application example, the unbound protein fraction was defined as a global parameter. Since it is also determined by the composition of the blood serum it can as such also be defined as individual parameter. However, the unbound fraction also depends on the lipophilicity of the drug, which is varied in our approach. Therefore, both parameters had to be sampled in the same MH block to consider the covariance between these parameters (Bernillon & Bois, 2000; Smith et al., 1992).

Advantages of using the highly-detailed mechanistic PBPK model were demonstrated by analyzing the example of pravastatin. Relationships between the physiological parameters were provided directly from the posterior and could be easily identified, for example a strong correlation was found between the enzyme activity of the MRP2 transporter and the interstitial permeability in all patients. This results from a contrary transport of pravastatin in the gastrointestinal tract, because MRP2 transports pravastatin back into the intestinal lumen. Therefore, by the analysis of the posterior, structural information about the model can be inferred.

Furthermore, beside the derivation of structural information about the PBPK model the identification of clinically relevant subgroups within the population is possible. By investigating the logarithmic mean values of the single patients with a Shapiro-Wilk test the assumption of more than one homogenous group was confirmed for OATP1B1. Additionally, the two groups of patients were assigned to different homozygous genotypes. This demonstrates the ability of our approach to make physiological inferences with very little prior information and only few individuals. The heterozygous genotype could not be assigned to an own group. However, Niemi et al. also showed that a significant separation of the heterozygous genotype is not possible (Niemi et al., 2006). Notably, the separation of different subgroups itself may also be possible with smaller models. However, the use of a mechanistic PBPK model can point out the relation between subgroup and genotype which makes our Bayesian-PBPK approach a suitable alternative to rather phenomenological methods (Link et al., 2008).

### Conclusions

Altogether, our presented Bayesian-PBPK approach provides many opportunities for the assessment of inter-individual variability in groups of patients. The advantages of MCMC compared to classical population PK lie in its usability even when only little prior information or experimental data is available. Especially for early phases of clinical development the identification of subgroups as well as special physiological properties can increase both patient safety and the level of information about the benefit-risk profile of a new drug candidate. The full physiological PBPK models allow the inference of physiological, drug-specific and genotype-specific knowledge separately, which therefore bears large extrapolation capacity to both, other drug candidates and populations. This will be greatly supported by the creation of a large database, where posterior knowledge about physiological parameter distributions can be collected iteratively. This would allow the consideration of posterior distributions of former Bayesian-PBPK runs as prior information in new runs, providing a framework for improving a mechanistic understanding of drug action, inter-individual variability and genotype-phenotype correlations with the help of growing amounts of different drugs or different populations.

## Electronic supplementary material

Additional file 1: Figure S1: Schematic representation of the enterohepatic circulation and the key transporting enzymes in pravastatin pharmacokinetics. It has to be noted, that this is only a simplified consideration for a better representation of the processes. However, the enterohepatic cycle and the transporting enzymes are integrated into the mechanistic whole-body physiologically-based pharmacokinetic model. (PDF 72 KB)

Additional file 2: Table S1: Parameter start values used for the initialization of the combined Bayesian-PBPK approach. (CSV 507 bytes)
