# Supplementary material for: Using Bayesian-PBPK modeling for assessment of inter-individual variability and subgroup stratification
Source: In Silico Pharmacol. 2013 Apr 11;1:6. doi: 10.1186/2193-9616-1-6 (PMC4230716; doi:10.1186/2193-9616-1-6)
Supplement: Supplementary file 1 — Additional file 1: Figure S1: Schematic representation of the enterohepatic circulation and the key transporting enzymes in pravastatin pharmacokinetics. It has to be noted, that this is only a simplified consideration for a better representation of the processes. However, the enterohepatic cycle and the transporting enzymes are integrated into the mechanistic whole-body physiologically-based pharmacokinetic model. (PDF 72 KB) [file 40203_2013_6_MOESM1_ESM.pdf]

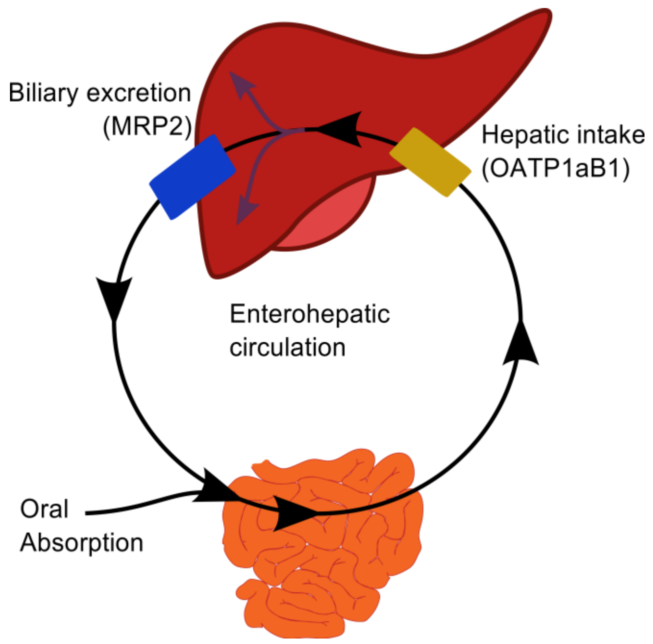


Supplementary Figure S1. Schematic representation of the enterohepatic circulation and the key transporting enzymes in pravastatin pharmacokinetics. It has to be noted, that this is only a simplified consideration for a better representation of the processes. However, the enterohepatic cycle and the transporting enzymes are integrated into the mechanistic whole-body physiologically-based pharmacokinetic model.
